# Burden of kidney cancer and attributed risk factors in China from 1990 to 2019

**DOI:** 10.3389/fpubh.2022.1062504

**Published:** 2022-12-16

**Authors:** Zongping Wang, Le Wang, Song Wang, Liping Xie

**Affiliations:** ^1^The First Affiliated Hospital, Zhejiang University School of Medicine (FAHZU), Hangzhou, Zhejiang, China; ^2^The Cancer Hospital of the University of Chinese Academy of Sciences (Zhejiang Cancer Hospital), Institute of Basic Medicine and Cancer (IBMC), Chinese Academy of Sciences, Hangzhou, Zhejiang, China

**Keywords:** kidney cancer, risk factor, burden of disease, epidemiology, trends

## Abstract

**Background:**

The changing trends and risk-attributed burdens of kidney cancer in China are unknown. Therefore, this study aimed to describe the latest status and trends of kidney cancer burden in China and its associated risk factors.

**Methods:**

The absolute numbers and rates of the incidence, deaths, and disability-adjusted life-years (DALYs) of kidney cancer in China were extracted from the Global Burden of Disease 2019 platform. Overall burden and burden attributed to smoking and high body mass index (BMI) were described. Average annual percent change (AAPC) was calculated to describe trend analyses from 1990 to 2019 using the Joinpoint regression program.

**Results:**

In 2019, 59,827 new cases, 23,954 deaths, and 642,799 DALYs of kidney cancer occurred in China, of which men accounted for 71.1, 70.5, and 72.0%, and the population aged ≥55 years accounted for 58.9, 77.9, and 60.1%, of new cases, deaths, and DALYs, respectively. From 1990 to 2019, the age-standardized incidence rate (per 100,000 person-years) increased from 1.16 in 1990 to 3.21 in 2019, with an AAPC of 3.4% (95% confidence interval [CI]: 3.1–3.8%, *p* < 0.05); the mortality rate increased from 0.70 to 1.27, with an AAPC of 2.1% (1.5–2.3%, *p* < 0.05); and the DALY rate increased from 0.70 to 1.27, with an AAPC of 2.1% (1.5–2.3%, *p* < 0.05). In 2019, the proportions of DALYs attributed to smoking and high BMI were 18.0% and 11.1%, respectively, and the DALY rates attributed to both smoking and high BMI increased from 1990 to 2019, with AAPC of 2.9% (2.6–3.3%, *p* < 0.05) and 4.8% (4.2–5.4%, *p* < 0.05), respectively.

**Conclusion:**

The kidney cancer burden in China has continued to grow over the recent three decades, with a severe burden among older adults and men. Therefore, timely preventive interventions for modifiable risk factors are required.

## 1. Introduction

Kidney cancer is a common urological cancer, and the incidence and mortality are ranked 15th of all cancers globally. According to estimates from the International Agency for Research on Cancer, 431,288 new cases and 179,368 deaths were reported worldwide in 2020 (1). Generally, the burden of kidney cancer differs from the sociodemographic index, which is commonly higher in countries with higher sociodemographic indexes than countries with lower sociodemographic indexes (2–5). In addition, the incidence and mortality of kidney cancer have been decreasing in a few developed countries and increasing in many developing countries over the recent three decades (3–7). In China, the incidence and mortality of kidney cancer remain relatively low, with 73,587 new cases and 43,196 deaths occurring annually (8). Notably, the 5-year relative survival rate of kidney cancer improved from 62.0% in 2003–2005 to 69.8% in 2012–2015 in China (9); however, it was still lower than that in developed countries (10).

Established risk factors for kidney cancer include age, cigarette smoking, excess body weight, hypertension, and familial cancer syndromes (2, 5). The risk of kidney cancer increased by 39% in current smokers and 20% in former smokers compared with that in never smokers (11). A positive dose–effect relationship of body mass index (BMI) was demonstrated in previous studies, and the relative risk increased by 25% per 5 kg/m^2^ increase in BMI (12). However, a few new risk factors with potentially causal relationships have been identified in recent studies, including trichloroethylene exposure, diabetes mellitus, and chronic kidney disease (2, 5, 13, 14). With rapid urbanization and transition to a Western diet and lifestyle in China, people experienced an increasing exposure to risk factors for kidney cancer, with a substantial increase in the prevalence of overweight and obesity in adults from 20% in 1992 to 42% in 2010 to 2012 (15), the prevalence of diabetes mellitus from 10.9% in 2013 to 12.4% in 2018 (16), and a stable but still high prevalence of cigarette smoking (17). However, the temporal trends in kidney cancer burden remain unknown.

Thus, in this study, we aimed to describe the latest burden and estimate the long-term trends in the incidence, mortality, and disability-adjusted life-years (DALYs) of kidney cancer in China and estimate the attributed burden of kidney cancer from cigarette smoking and excess body weight, which are supposed to be useful for decision-making on designing primary and secondary prevention strategies for kidney cancer.

## 2. Methods

### 2.1. Data source

The Global Burden of Diseases 2019 (GBD 2019) estimated the burden and attributed risk factors of 369 diseases and injuries in 204 countries and territories, and input data were extracted from censuses, household surveys, civil registration and vital statistics, disease registries, health service use, disease notifications, and other sources (18). We used the data to estimate the trends of kidney cancer disease burden in China from 1990 to 2019, including the following measurement indicators: deaths, incidence, and DALYs in the GBD 2019 platform (19). The DALYs represent a combined measure of health loss from both nonfatal and fatal outcomes, equal to years of life lost and years lived with a disability. Detailed methods for GBD have been previously reported (18, 20–22).

### 2.2. Definition of disease and risk factors

The International Classification of Diseases 10 (ICD-10) codes mapped to the GBD cause list for kidney cancer incidence and mortality are C64–C64.2, C64.4–C64.6, C64.8–C64.9, C65–C65.2, C65.9, D30.0–D30.1, and D41.0–D41.1 (22). A total of 87 risk factors and combinations of risk factors were estimated in GBD 2019, and the attributable number and age-standardized rate of deaths and DALYs by selected risk factors were estimated according to a comparative risk assessment (20, 23). For kidney cancer, the attributed burden of smoking, high BMI, and occupational exposure to trichloroethylene were available on the GBD platform, but occupational exposure to trichloroethylene was not included in the analysis owing to its relatively low absolute effects and rates. Smoking was defined as the current or former smoking of any tobacco product. Current smokers are individuals who use smoked tobacco products on a daily or occasional basis. Former smokers were individuals who quit using all smoked tobacco products for at least 6 months, where possible, or according to the definition used by the survey. A high BMI is defined as a BMI >20–25 kg/m^2^ for adults (20+ years of age). A high BMI for children (1–19 years of age) was defined as being overweight or obese based on the International Obesity Task Force standards.

### 2.3. Statistical analysis

All rates were reported per 100,000 person-years. Age-standardized rates were calculated according to the GBD World Population Standard (21). In GBD studies, the incident cases, death cases, and DALYs were simulated by using complicated models, which were quite different from the reported numbers from real-world cancer registration. The model used to simulate the GBD took the posterior distribution of each input data into consideration, which means that the output results in each simulation were different from others. Therefore, the 95% uncertainty intervals (UIs) were calculated by taking 1,000 samples from the posterior distribution of the respective step in the modeling process and reported as the 2.5th and 97.5th values for each estimate. Trends in the incidence, mortality, and DALY rates of kidney cancer in China from 1990 to 2019 were analyzed using the Joinpoint regression program. The average annual percent change (AAPC) was calculated for the entire period from 1900 to 2019, and the estimated annual percent change (APC) was calculated for each segment. The approximate 95% confidence intervals (CIs) for AAPC and APC were also calculated using the empirical quantile method. The two-tailed *t*-test was used for statistical inference, the null hypothesis of true AAPC or APC was 0, and the Bonferroni adjustment was used for multiple tests. A two-sided *p* < 0.05 was considered to be statistically significant. The Joinpoint regression analyses were conducted using the Joinpoint regression program version 4.9.0.0 (National Cancer Institute, Bethesda, USA).

## 3. Results

### 3.1. Current status of kidney cancer burden in 2019

**Figure 1:**
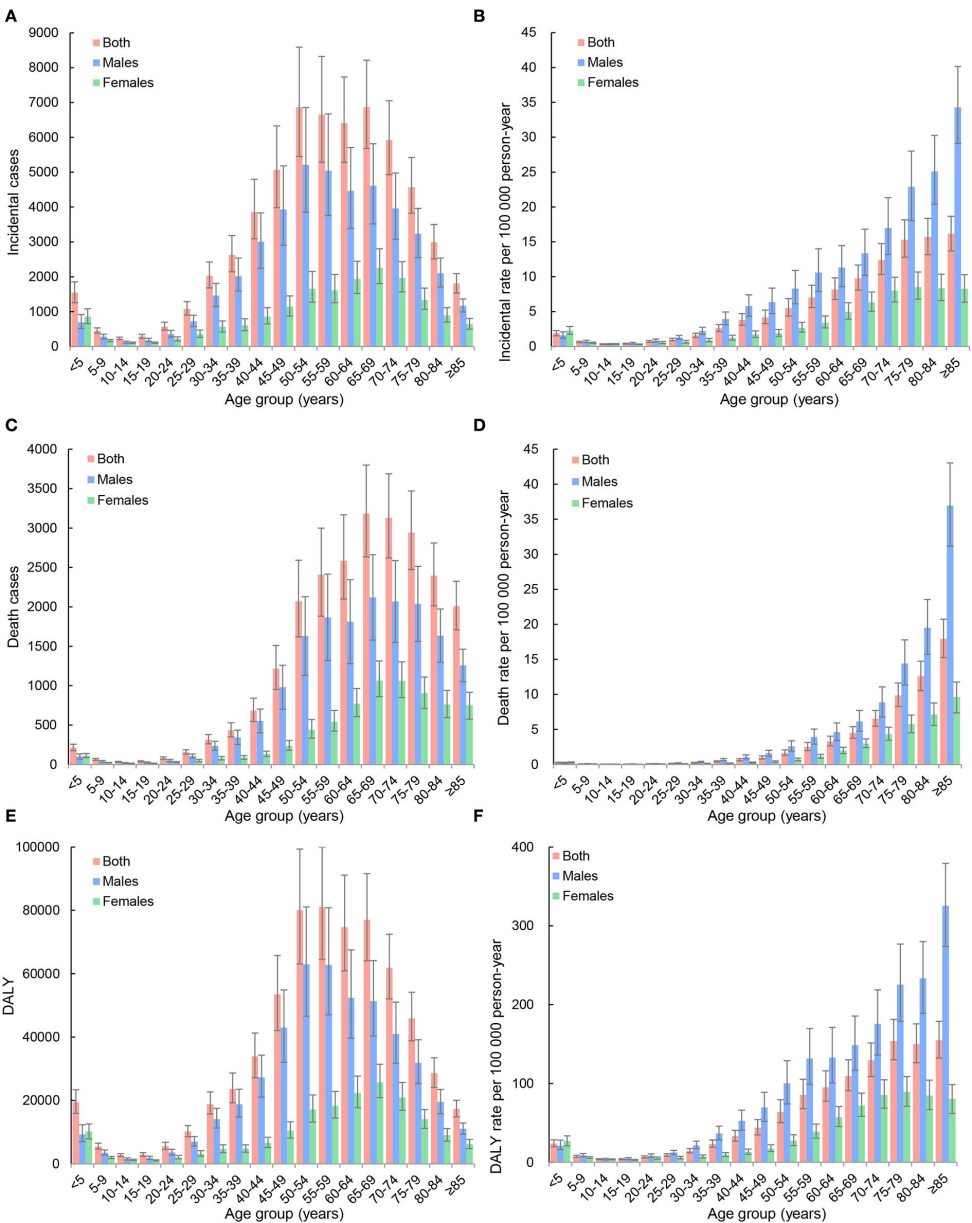
Age distributions of incidence, mortality, and disability-adjusted life-years (DALYs) of kidney cancer in China, 2019. **(A)** Incident cases; **(B)** incidence rates per 100,000 person-years; **(C)** death cases; **(D)** mortality rates per 100,000 person-years; **(E)** DALYs; **(F)** DALY rates per 100,000 person-years.

In 2019, 59,827 (95% UI: 49,506–71,238) new cases, 23,954 (19,766–28,481) deaths, and 642,799 (533,658–763,976) DALYs of kidney cancer occurred in China, accounting for 16.1% of new cases, 14.4% of deaths, and 15.9% of DALYs worldwide. The age-standardized incidence rate (ASIR), the mortality rate (ASMR), and the DALY rate (ASDR) were 3.21 (2.70–3.79), 1.68 (1.39–2.00), and 34.28 (28.95–40.16) per 100,000 person-years, respectively. Men had a higher risk of kidney cancer burden than women, and the ratios of the burden of men to women were 2.41 for ASIR, 2.30 for ASMR, and 2.47 for ASDR, respectively. Most kidney cancer burden occurred in the population aged ≥55 years, and the proportions were 58.9% for new cases, 77.9% for death cases, and 60.1% for DALYs. Detailed results are presented in Figure 1 and Table 1.

**Table 1:** Incidence, mortality, and disability-adjusted life-year (DALYs) of kidney cancer in China, 2019.

	**Incidence**		**Mortality**		**DALYs**	

	**No. of cases**	**Rate**	**No. of cases**	**Rate**	**No. of cases**	**Rate**
Global	371,747(344,594–402,350)	4.55 (4.22–4.93)	166,438 (155,461–176,302)	2.08(1.93–2.20)	4,052,817 (3,801,041–4,317,487)	49.62(46.46–52.94)
China	59,827(49,506–71,238)	3.21(2.70–3.79)	23,954 (19,766–28,481)	1.68(1.39–2.00)	642,799 (533,658–763,976)	34.28(28.95–40.16)
**Sex**						
Male	42,554(33,141–53,165)	4.63(3.67–5.73)	16,883 (13,178–20,842)	2.33(1.82–2.88)	462,869 (363,081–571,556)	49.65(39.57–60.57)
Female	17,273 (13,840–21,299)	1.92(1.58–2.32)	7071 (5680–8625)	1.01(0.81–1.24)	179,931 (147,905–216,685)	20.07(16.75–23.75)
**Age years**						
0–19	2,510(2,158–2,928)	0.84(0.72–0.98)	352 (304–409)	0.12(0.10–0.14)	30,568(26,129–35,619)	10.19(8.71–11.88)
20–54	22,087(17,700–26,825)	2.87(2.30–3.48)	4,952(3,938–6,066)	0.64(0.51–0.79)	225,636(181,930–273,804)	29.28(23.61–35.53)
≥55	35,230(29,408–41,774)	10.02(8.36–11.88)	18,650(15,500–21,983)	5.30(4.41–6.25)	386,595 (320,630–457,723)	57.85(47.98–68.49)

Data in the parentheses are 95% uncertainty intervals. The unit of rate was calculated as per 100,000 person-years, and adjusted by standardized age structure.

### 3.2. Temporal trends of kidney cancer burden from 1990 to 2019

**Figure 2:**
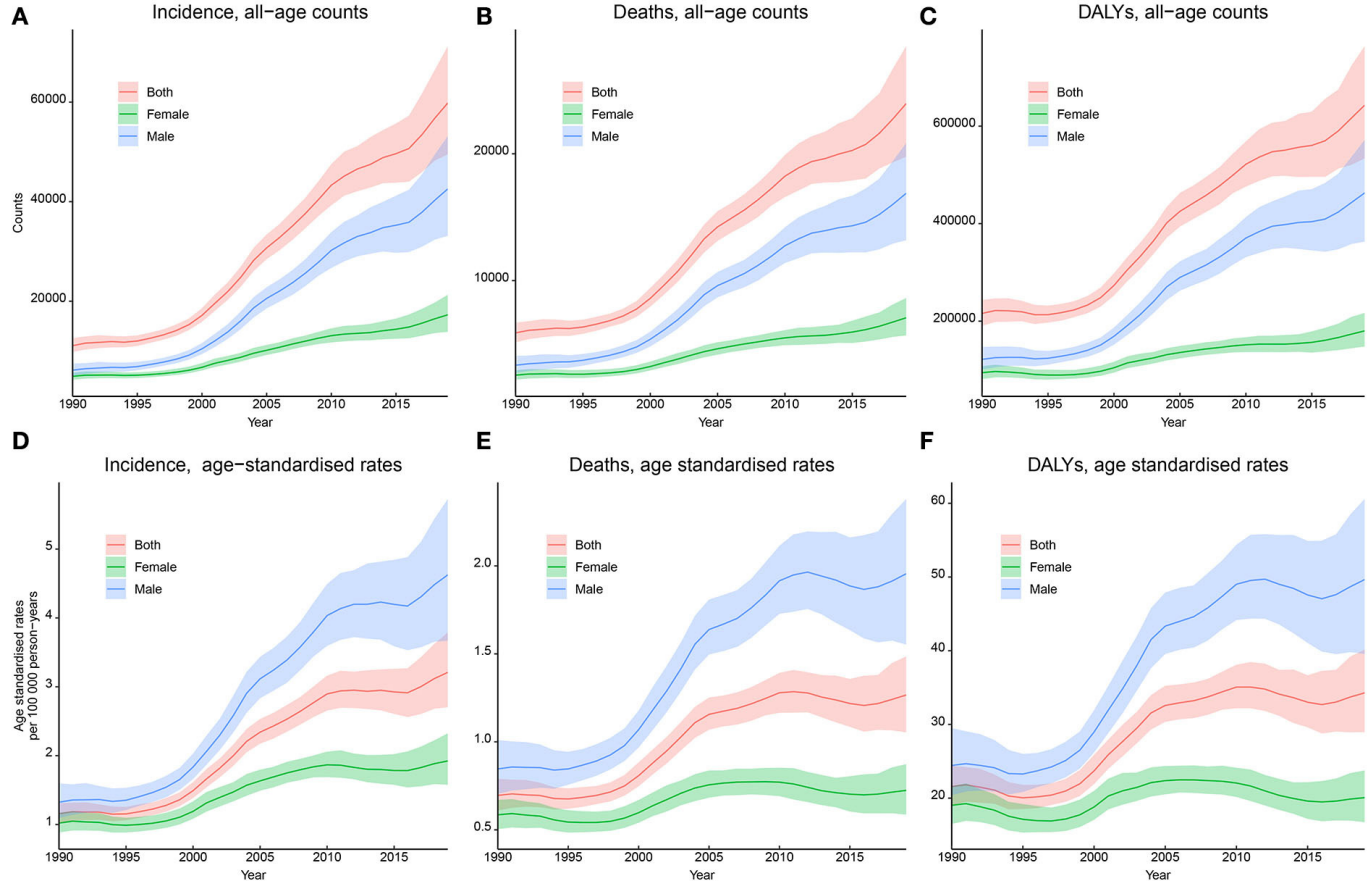
Trends in the incidence, mortality, and disability-adjusted life years (DALYs) of kidney cancer in China, 1990–2019. **(A)** Incident cases; **(B)** incidence rates per 100,000 person-years; **(C)** death cases; **(D)** mortality rates per 100,000 person-years; **(E)** DALYs; **(F)** DALY rates per 100,000 person-years.

In China, the incidence of kidney cancer increased from 11,072 (9,794–12,587) in 1990 to 59,827 (49,506–71,238) in 2019 (Figure 2), and the ASIR substantially increased from 1.16 (1.02–1.31) in 1990 to 3.21 (2.70–3.79) per 100,000 person-years in 2019, with an AAPC of 3.4% (95% CI: 3.1–3.8%, *p* < 0.05). Results of subgroup analyses showed that men contributed more to the increasing burden, with a proportion of the absolute number of incidental cases from 55.6% in 1990 to 71.1% in 2019 and an increasing male–female ratio of ASIR from 1.30 in 1990 to 2.41 in 2019 (**Table 3**). A more rapidly increasing trend (AAPC: 4.3%, 95% CI: 3.7–4.9%, *p* < 0.05) was found for ASIR in men than that for ASIR in women (AAPC: 2.2%, 95% CI: 1.7–2.7%, *p* < 0.05; Table 2).

**Table 2:** Temporal trends in the burden and risk-attributed DALY for kidney cancer in China from 1990 to 2019.

**Indicator**	**AAPC**	**Range**	**Trend 1**		**Trend 2**		**Trend 3**		**Trend 4**		**Trend 5**		**Trend 6**	
			**Year**	**APC**	**Year**	**APC**	**Year**	**APC**	**Year**	**APC**	**Year**	**APC**	**Year**	**APC**
**Incidence**														
Both	**3.4**	3.1 to 3.8	1990–1997	−0.2	1997–2004	**9.0**	2004–2010	**5.1**	2010–2016	−0.1	2016–2019	**3.3**		
Female	**2.2**	1.7 to 2.7	1990–1997	0	1997–2004	**6.8**	2004–2010	**2.9**	2010–2015	−1.2	2015–2019	**1.8**		
Male	**4.3**	3.7 to 4.9	1990–1998	**1.4**	1998–2004	**11.9**	2004–2010	**5.6**	2010–2019	**1.3**				
**Death**														
Both	**2.1**	1.5 to 2.3	1990–1999	0.3	1999–2005	**8.9**	2005–2012	**1.2**	2012–2017	−2	2017–2019	4.8		
Female	0.3	−0.4 to 1.0	1990–1997	**−3.1**	1997–2006	**5.6**	2006–2019	**−1.5**						
Male	**3.0**	2.0 to 4.0	1990–1992	5.9	1992–1995	−3.9	1995–1998	4.2	1998–2004	**9.2**	2004–2010	**3.3**	2010–2019	0.2
**DALY**														
Both	**1.5**	1.2 to 1.9	1990–1995	−1.8	1995–1998	1.0	1998–1904	**7.5**	2004–2011	**1.5**	2011–2016	**−1.6**	2016–2019	**1.8**
Female	0.1	−0.2 to 0.4	1990–1997	−2.3	1997–2003	**4.7**	2003–2008	0.8	2008–2016	**−2**	2016–2019	1.3		
Male	**2.4**	2.1 to 2.8	199019–96	**−0.9**	1996–1999	**4.3**	1999–2004	**9.5**	2004–2011	**2.6**	2011–2016	**−1.2**	2016–2019	**1.7**
**DALY attributed to smoking**														
Both	**2.9**	2.6 to 3.3	1990–1995	**1.4**	1995–1999	**4.3**	1999–2004	**10.4**	2004–2012	**1.5**	2012–2016	**−2.1**	2016–2019	**2.4**
Female	**1.6**	0.7 to 2.5	1990–1996	0.1	1996–2005	**8.2**	2005–2019	**−1.8**						
Male	**3.0**	2.7 to 3.4	1990–1996	**1.7**	1996–1999	**4.9**	1999–2004	**10.2**	2004–2012	**1.8**	2012–2016	**−1.9**	2016–2019	**2.3**
**DALY attributed to high body-mass index**														
Both	**4.8**	4.2 to 5.4	1990–1995	0.1	1995–2000	**7.2**	2000–2004	**11.9**	2004–2011	**5.1**	2011–2017	**1.3**	2017–2019	**7.0**
Female	**3.3**	2.7 to 3.8	1990–1994	−0.1	1994–1998	**3.4**	1998–2005	**7.7**	2005–2010	**2.8**	2010–2015	0.2	2015–2019	**3.3**
Male	**6.1**	5.3 to 6.9	1990–1997	**2.1**	1997–2004	**13.4**	2004–2011	**6.8**	2011–2016	1.3	2016–2019	**5.6**		

Numbers in bold indicate statistical significance. All rates were age-standardized and temporal trends were calculated by using the Join-point regression. AAPC, annual average percentage of change; APC, annual percentage of change; DALY, disability-adjusted life-year.

For kidney cancer mortality, the absolute number of death cases kept increasing from 5,880 (5,137–6,688) in 1990 to 23,954 (19,766–28,481) in 2019, and the ASMR substantially increased from 0.70 (0.61–0.79) in 1990 to 1.27 (1.05–1.49) per 100,000 person-years in 2019, with an AAPC of 2.1% (1.5–2.3%, *p* < 0.05). Subgroup analyses showed that the proportion of the absolute number of men increased from 56.6% in 1990 to 70.5% in 2019, and the male–female ratio of ASMR increased from 1.45 in 1990 to 2.70 in 2019 (Table 3). A significant increasing trend was found for ASMR in men from 1990 to 2019 (AAPC: 3.0%, 2.0–4.0%, *p* < 0.05), whereas a stable trend was found for ASMR in women (AAPC: 0.3%, −0.4 to 1.0%, *p* > 0.05), as shown in Figure 2 and Table 2.

**Table 3:** Trends in the men's proportion for absolute numbers and male–female ratio for rates of kidney cancer.

	**Men's proportion for absolute numbers**			**Male-female ratio for rates**		
**Year**	**Incidence**	**Mortality**	**DALYs**	**Incidence**	**Mortality**	**DALYs**
1990	55.6%	56.6%	56.3%	1.30	1.45	1.28
1991	55.6%	56.7%	56.4%	1.29	1.45	1.28
1992	56.1%	57.0%	56.8%	1.31	1.46	1.30
1993	56.5%	57.3%	57.3%	1.32	1.48	1.31
1994	56.7%	57.9%	57.5%	1.34	1.51	1.33
1995	57.2%	58.7%	58.1%	1.37	1.56	1.36
1996	58.0%	59.4%	58.9%	1.41	1.60	1.40
1997	58.7%	60.1%	59.6%	1.44	1.64	1.43
1998	59.2%	60.8%	60.1%	1.47	1.69	1.46
1999	59.9%	61.5%	60.9%	1.50	1.73	1.49
2000	60.8%	62.5%	61.8%	1.54	1.79	1.54
2001	61.5%	63.4%	62.6%	1.57	1.85	1.57
2002	63.0%	64.4%	64.1%	1.65	1.92	1.66
2003	64.8%	65.6%	65.9%	1.75	2.00	1.77
2004	66.2%	66.9%	67.3%	1.85	2.11	1.88
2005	67.0%	67.5%	68.1%	1.91	2.17	1.94
2006	67.2%	67.6%	68.4%	1.92	2.18	1.96
2007	67.5%	67.8%	68.8%	1.94	2.21	1.98
2008	68.1%	68.4%	69.4%	1.99	2.28	2.05
2009	68.9%	69.1%	70.1%	2.07	2.37	2.13
2010	69.8%	69.9%	71.0%	2.16	2.48	2.22
2011	70.3%	70.4%	71.5%	2.22	2.56	2.29
2012	70.9%	70.9%	72.1%	2.29	2.65	2.37
2013	71.1%	71.0%	72.3%	2.33	2.69	2.41
2014	71.2%	71.0%	72.3%	2.35	2.70	2.43
2015	71.0%	70.7%	72.1%	2.35	2.68	2.43
2016	70.8%	70.4%	71.8%	2.34	2.67	2.41
2017	70.8%	70.4%	71.8%	2.36	2.68	2.43
2018	71.0%	70.4%	71.9%	2.38	2.68	2.45
2019	71.1%	70.5%	72.0%	2.41	2.70	2.47

For DALYs, the absolute number continued to increase from 215,763 (190,358–243,561) in 1990 to 642,799 (533,658–763,976) in 2019, and the ASDR substantially increased from 21.59 (19.04–24.40) per 100,000 person-years in 1990 to 34.28 (28.95–40.16) per 100,000 person-years in 2019, with an AAPC of 1.5% (1.2–1.9%, *p* < 0.05). Subgroup analyses showed that the proportion of the absolute number of men increased from 56.3% in 1990 to 72.0% in 2019, and the male–female ratio of ASDR from 1.28 in 1990 to 2.47 in 2019 (Table 3). A significant increasing trend was found for ASDR in men from 1990 to 2019 (AAPC: 2.4%, 2.1–2.8%, *p* < 0.05), whereas a stable trend was found for ASDR in women (AAPC: 0.1%, −0.2 to 0.4%, *p* > 0.05), as shown in Figure 2 and Table 2.

### 3.3. Temporal trends of the risk-attributed burden of kidney cancer from 1990 to 2019

**Figure 3:**
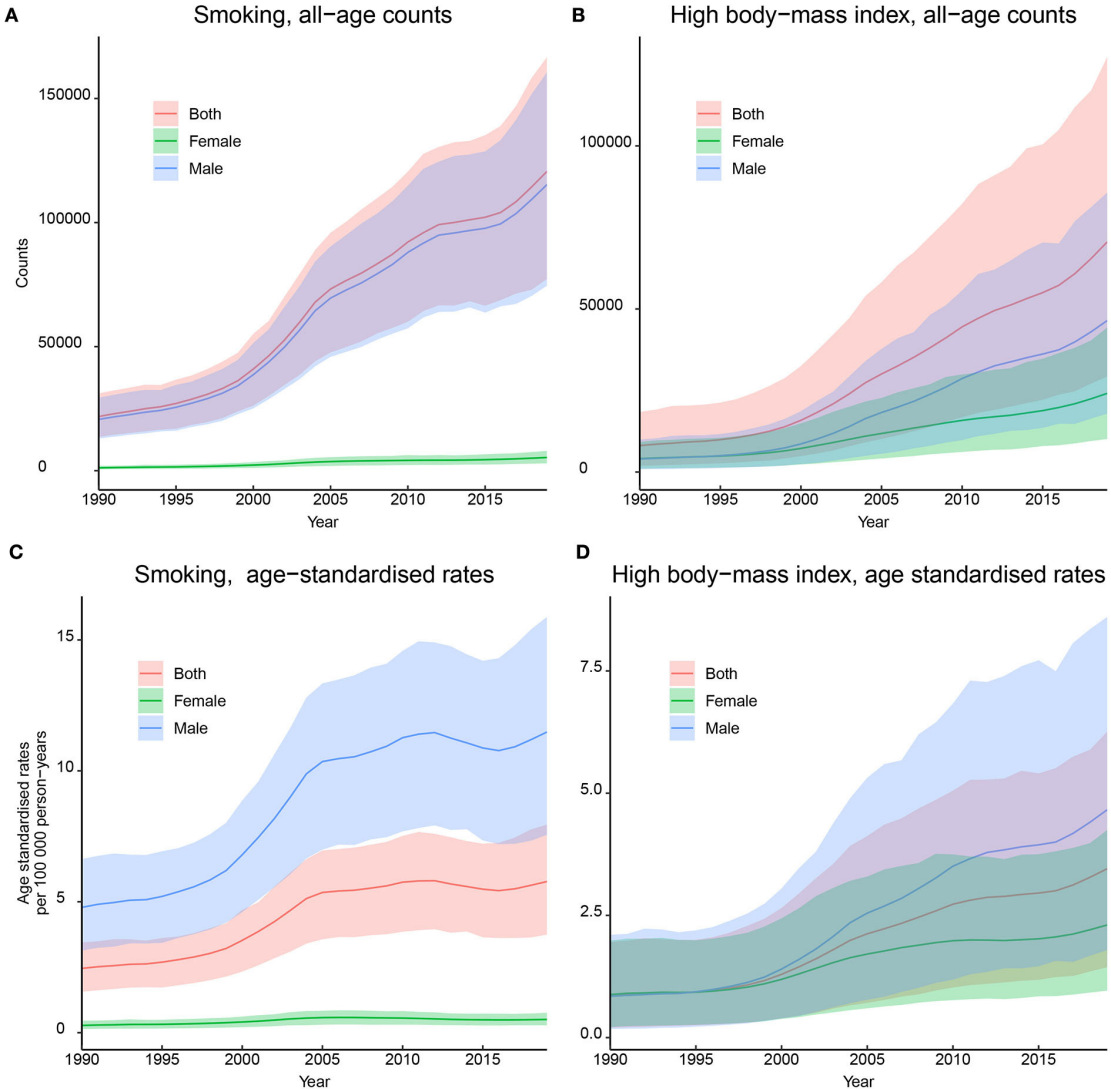
Trends in the disability-adjusted life-years (DALYs) of kidney cancer attributed by smoking and high body mass index (BMI) in China, 1900–2019. **(A)** DALYs attributed by smoking; **(B)** DALYs attributed by high BMI; **(C)** DALY rates attributed by smoking; **(D)** DALY rates attributed by high BMI. All rates were calculated as per 100,000 person-years.

The absolute DALYs attributed to smoking substantially increased from 21,840 (13,578–31,192) in 1990 to 120,620 (77,266–166,681) in 2019, and the absolute DALYs attributed to high BMI increased from 8,117 (1,906–18,424) in 1990 to 70,544 (29,312–127,229) in 2019. The ASDRs attributed to smoking and high BMI showed increasing trends, and the AAPCs were 2.9% (2.6–3.3%, *p* < 0.05) and 4.8% (4.2–5.4%, *p* < 0.05), respectively. Subgroup analyses by sex showed similar increasing trends, and the AAPCs of ASDR in men were higher than those in women. The details are presented in Figure 3 and Table 2.

## 4. Discussion

In this study, we comprehensively explored the long-term changing trends and risk-attributed burden of kidney cancer in China based on the GBD platform. Although the age-standardized burden of kidney cancer in China is lower than that of the average burden worldwide, ASIR, ASMR, and ASDR in China have continued to increase over the recent three decades. The burden of kidney cancer increased with age, with a severe burden among the older adult population, and sex differences were noteworthy. In addition, the burden attributed to smoking and high BMI has continued to increase. The current results are fundamental to guiding further prevention policymaking and reinforcement of interventions for kidney cancer in China.

During the recent three decades, both the absolute numbers and age-standardized rates of incidence, mortality, and DALYs of kidney cancer have been increasing in China, posing a health threat. Although the age-standardized rates in China were lower than that of the global average, the large population made a tremendous absolute number, which is supported by previous studies (4, 6). Meanwhile, 59% of new kidney cancer cases, 78% of death cases, and 60% of DALYs occurred among the population aged ≥55 years in China, which is similar to the global level (4). By 2022, there were >260 million people aged >60 years in China, and this number is projected to double by 2050 (24). Thus, the visible growth of the aging population will result in a continuously increasing trend of kidney cancer and an enormous public health burden. Notably, the increasing trends of the mortality rate were slower than that of the incidence rate. There is no doubt that the rapidly increasing trends in the incidence rate would result in more prevalent cases of kidney cancer since the mortality rate was a combined indicator of incidence rate and survival rate. In the past decades, immune checkpoint inhibitors and vascular endothelial growth factor receptor (VEGFR)-targeting tyrosine kinase inhibitors have shown efficacy in the treatment of metastatic renal cell carcinoma (25), and population-based surveillance data indicated that the 5-year relative survival rate of kidney cancer in China has been improved from 62.0% in 2003–2005 to 69.8% in 2012–2015 (9). Therefore, the slowing speed in the increase of mortality was predominantly achieved by improving treatment methods.

Based on available information from the GBD platform, cigarette smoking and high BMI were two major modifiable risk factors for kidney cancer in China, accounting for 18.8% and 11.0% of all DALYs, respectively, which was similar to previous studies (4, 23). However, the continuously increasing trends of kidney cancer burden attributed to smoking and high BMI are notable for China, which is quite different from the United States, where the burden of kidney cancer has kept decreasing and the prevalence of smoking and high BMI has been effectively reduced (26). The rising burden of kidney cancer could be partly explained by the increasing prevalence of high BMI in China. Data from the China National Nutrition Surveys showed that among adults (aged ≥ 18 years), the mean BMI increased from 21.9 kg/m^2^ in 1992 to 22.6 kg/m^2^ in 2002 and 23 kg/m^2^ in 2010–2012, and the prevalence of overweight and obesity among adults increased from 20.6% in 1991 to 29.9% in 2002 and 41.3% in 2010–2012 (15). However, the increase in kidney cancer attributable to smoking cannot be fully explained. According to reports from serial cross-sectional national health service surveys, the standardized smoking prevalence in China is consistently high, with a proportion of current smokers of 26.0% in 2003, 24.9% in 2008, and 25.2% in 2013. Male smoking prevalence was maintained at approximately 47%, while female smoking prevalence remained at a very low rate (nearly 3%) (17). A few more potential risk factors must be considered, such as hypertension (27), diabetes mellitus (16), chronic kidney disease (28), and alcohol consumption (29), whereas the corresponding kidney cancer burden attributed to these risk factors could not be quantitatively estimated and analyzed, owing to the data availability of the GBD 2019.

Our study found that sex discrepancy existed for kidney cancer in China, with the male–female ratio age-standardized rate at 2.41 for incidence, 2.30 for mortality, and 2.47 for DALY, which exceeded the global male–female rate ratios (4, 30). The underlying reasons for this sex discrepancy are unknown, but sex differences in the prevalence of attributed risk factors play a vital role (16, 17, 27–29, 31), especially the large gap in smoking prevalence (47.2% in men vs. 2.7% in women) (17). In addition, sex differences in genomic characteristics might explain more. A few studies have demonstrated sex as an independent factor for the progression and survival of kidney cancer (30, 32–34). Thus, when implementing population-based prevention strategies and providing patient-level treatment regimens for kidney cancer, sex differences should be considered.

Our study has some limitations, and readers should be cautious when interpreting our results. First, data from the GBD platform were estimated from complex analytical models by integrating multiple sources, including surveillance data, surveys, and publications, which are quite different from the results reported from actual cancer registries in China. Second, the current GBD 2019 platform only provides the estimated kidney cancer burden attributed to smoking, high BMI, and occupational exposure to trichloroethylene, and a few more risk factors have been proposed, but the attributed cancer burden could not be quantitatively measured. Despite these limitations, the GBD platform has continuously extended its original data source, advanced modeling strategies, and enriched estimation outputs over the past decades. Based on data with the most up-to-date and long-term robust trends, our study provides a comprehensive understanding of the overall and risk-attributed burden of kidney cancer in China.

## 5. Conclusion

In this study, we performed a comprehensive analysis to assess the up-to-date burden of kidney cancer in China from 1990 to 2019. The incidence, mortality, and DALYs of kidney cancer have increased over the recent three decades. In general, the burden of kidney cancer increases with age, with a severe burden among the older adult population, and sex differences are noteworthy. In addition, the burden attributed to smoking and a high BMI has continued to increase in China. With the rapid acceleration of urbanization and an aging population, it is important to be aware of the potentially increased burden of kidney cancer. Reinforcing a healthy lifestyle for the public would help minimize the burden of kidney cancer in the future.

## Data availability statement

The raw data supporting the conclusions of this article will be made available by the request from the corresponding author.

## Author contributions

ZW and LX: conception and design. ZW, LW, and SW: acquisition, analysis, or interpretation of data. ZW: drafting of the manuscript. LW, SW, and LX: critical revision of the manuscript for important intellectual content. LW: statistical analysis. LX: administrative, technical, or material support, and supervision. All authors contributed to the article and approved the submitted version.
